# Medical Journal in Havana

**Published:** 1843-06

**Authors:** 


### MEDICAL JOURNAL IN HAVANA.

Our readers may not all know that the profession in the island of Cuba has an organ, through which its members communicate with each other and with their brethren abroad. We have recently received, in exchange, the "*Repertorio Medico-Habanero,*" published, as its name imports, at Havana, in the Spanish language. "The "Repertorio" is a semi-monthly, of sixteen large octavo pages, double columns; Dr. Gutierrez is the chief editor, or "Director," assisted by Drs. Miranda, Lanuza, Costales, and Chamorro. A large part of its contents is original, and it seems to be conducted with considerable spirit. In one of the numbers received by us, we observe a very flattering notice of Professor Gross' "Elements of Pathological Anatomy." C.

